# Switch from a ritonavir to a cobicistat containing antiretroviral regimen and impact on tacrolimus levels in a kidney transplant recipient

**DOI:** 10.1186/s12985-023-02058-3

**Published:** 2023-05-05

**Authors:** Andrea Erba, Catia Marzolini, Katharina Rentsch, Marcel Stoeckle, Manuel Battegay, Michael Mayr, Maja Weisser

**Affiliations:** 1 Division of Infectious Diseases and Hospital Epidemiology, University Hospital Basel, University Basel, Petersgraben 4, 4031 Basel, Switzerland; 2 Department of Molecular and Clinical Pharmacology, University of Liverpool, Liverpool, UK; 3 Department of Clinical Chemistry and Laboratory Medicine, University Hospital Basel, Basel, Switzerland; 4 Medical Outpatient Department, University Hospital Basel, Basel, Switzerland

**Keywords:** HIV, Kidney transplantation, Drug–drug interaction, Cobicistat, Ritonavir, Pharmacokinetic booster

## Abstract

**Background:**

Solid-organ transplantation due to end-stage organ disease is increasingly performed in people living with HIV. Despite improved transplant outcomes, management of these patients remains challenging due to higher risk for allograft rejection, infection and drug–drug interactions (DDIs). Complex regimens for multi-drug resistant HIV-viruses may cause DDIs particularly if the regimen contains drugs such as ritonavir or cobicistat.

**Case presentation:**

Here we report on a case of an HIV-infected renal transplant recipient on long-term immunosuppressive therapy with mycophenolate mofetil and tacrolimus dosed at 0.5 mg every 11 days due to the co-administration of a darunavir/ritonavir containing antiretroviral regimen. In the presented case the pharmacokinetic booster was switched from ritonavir to cobicistat for treatment simplification. A close monitoring of tacrolimus drug levels was performed in order to prevent possible sub- or supratherapeutic tacrolimus trough levels. A progressive decrease in tacrolimus concentrations was observed after switch requiring shortening of tacrolimus dosing interval. This observation was unexpected considering that cobicistat is devoid of inducing properties.

**Conclusions:**

This case highlights the fact that the pharmacokinetic boosters ritonavir and cobicistat are not fully interchangeable. Therapeutic drug monitoring of tacrolimus is warranted to maintain levels within the therapeutic range.

**Supplementary Information:**

The online version contains supplementary material available at 10.1186/s12985-023-02058-3.

## Introduction

The prevalence of chronic and end-stage kidney disease in people living with HIV (PLWH) is increasing with the improved life expectancy and the related aging of the HIV population [1, 2]. As a consequence, kidney transplantation is increasingly performed in PLWH. The management of these patients is challenged by an increased risk for allograft rejection and drug–drug interactions (DDI) particularly between boosted antiretroviral regimens and immunosuppressive drugs [3–5]. The development of new antiretroviral drugs, notably un-boosted integrase strand transfer inhibitors (INSTI), has considerably reduced DDIs with calcineurin or mTOR inhibitors [3, 6]. However, DDIs remain a problem in individuals with multidrug-resistant HIV, who consequently need more complex antiretroviral treatments combining multiple antiretroviral drug classes including protease inhibitors (PI). PIs require a pharmacokinetic booster in order to achieve sufficient concentrations to inhibit viral replication. The pharmacokinetic booster ritonavir is a potent inhibitor of the cytochrome P450 (CYP) 3A4 isoenzyme and therefore has a high potential to cause DDIs—notably with immunosuppressants. For instance, ritonavir was shown to increase the exposure of the sensitive CYP3A4 substrate tacrolimus by 57-fold and its elimination half-life by sevenfold [7]. The management of this interaction requires both a substantial reduction in tacrolimus dose and an extension of the dosing interval [8]. The alternative pharmacokinetic booster cobicistat (on the market since 2012) is also a potent inhibitor of CYP3A4, but is a more selective CYP inhibitor and is devoid of inducing properties [9–11]. Thus, switching from a pharmacokinetic booster to another could potentially impact the exposure of immunosuppressive drugs and the dosage requirement. Here, we report on the DDI by closely monitoring tacrolimus levels in a kidney transplant recipient living with HIV who was switched from a ritonavir- to a cobicistat-containing antiretroviral regimen.

## Case

A 55-year-old man diagnosed with HIV in 1995 suffered from progressive chronic kidney disease caused by biopsy-proven HIV-associated focal segmental glomerulosclerosis. As described in a previous publication [8], he started hemodialysis in July 2006 and received a deceased-donor kidney transplant in September 2007. The patient had received multiple different antiretroviral regimens in the past, including didanosine (nucleoside reverse transcriptase inhibitor (NRTI)), saquinavir (PI) boosted with ritonavir in 1997, then sequentially switched to different PIs (nelfinavir, indinavir, amprenavir, lopinavir), and later to efavirenz (non-nucleoside reverse transcriptase inhibitor (NNRTI)), with abacavir (NRTI), lamivudine (NRTI), zidovudine (NRTI) and stavudine (NRTI) as backbone drugs. Most switches were due to HIV drug resistance mutations. At the time of kidney transplantation in 2007, the patient had a persistent low-level viremia under the combined treatment with didanosine, lamivudine, efavirenz and the fusion inhibitor enfuvirtide. Resistance testing at that time showed multiple acquired high-level HIV drug resistance mutations against all NRTIs and PIs with the exception of darunavir (PI). Therefore, a new HIV antiretroviral salvage therapy with raltegravir (INSTI) and etravirine (NNRTI) combined with tenofovir disoproxil (NRTI) and darunavir (PI) boosted with ritonavir was started. The DDI between ritonavir-boosted darunavir (darunavir/r) and the calcineurin-inhibitor tacrolimus has been described in detail previously [8]. Briefly, the potent inhibition of CYP3A4 by darunavir/r required a substantial reduction in tacrolimus dosage from 4 mg twice daily to 0.5 mg once weekly to achieve trough levels of 6–7 µg/L with further extension of the dosing interval to every 11 days to maintain stable trough levels of 3–5 µg/L in long-term follow-up. At a later time, the chemokine receptor 5 (CCR5) antagonist maraviroc was added and tenofovir disoproxil fumarate (NRTI) was replaced by tenofovir alafenamide (NRTI). This antiretroviral treatment was continued until 2020, allowing a constant viral load suppression. During the course of the transplantation, the patient did not experience any sign of rejection based on biopsies at month 3 and 6, and at year 4 and 7 post-transplantation. The main issue has been the development of a post-transplant diabetes mellitus complicated by peripheral neuropathy, peripheral arterial occlusive disease, diabetic foot syndrome with multiple orthopedic interventions and episodes of erysipelas.

**Fig. 1:**
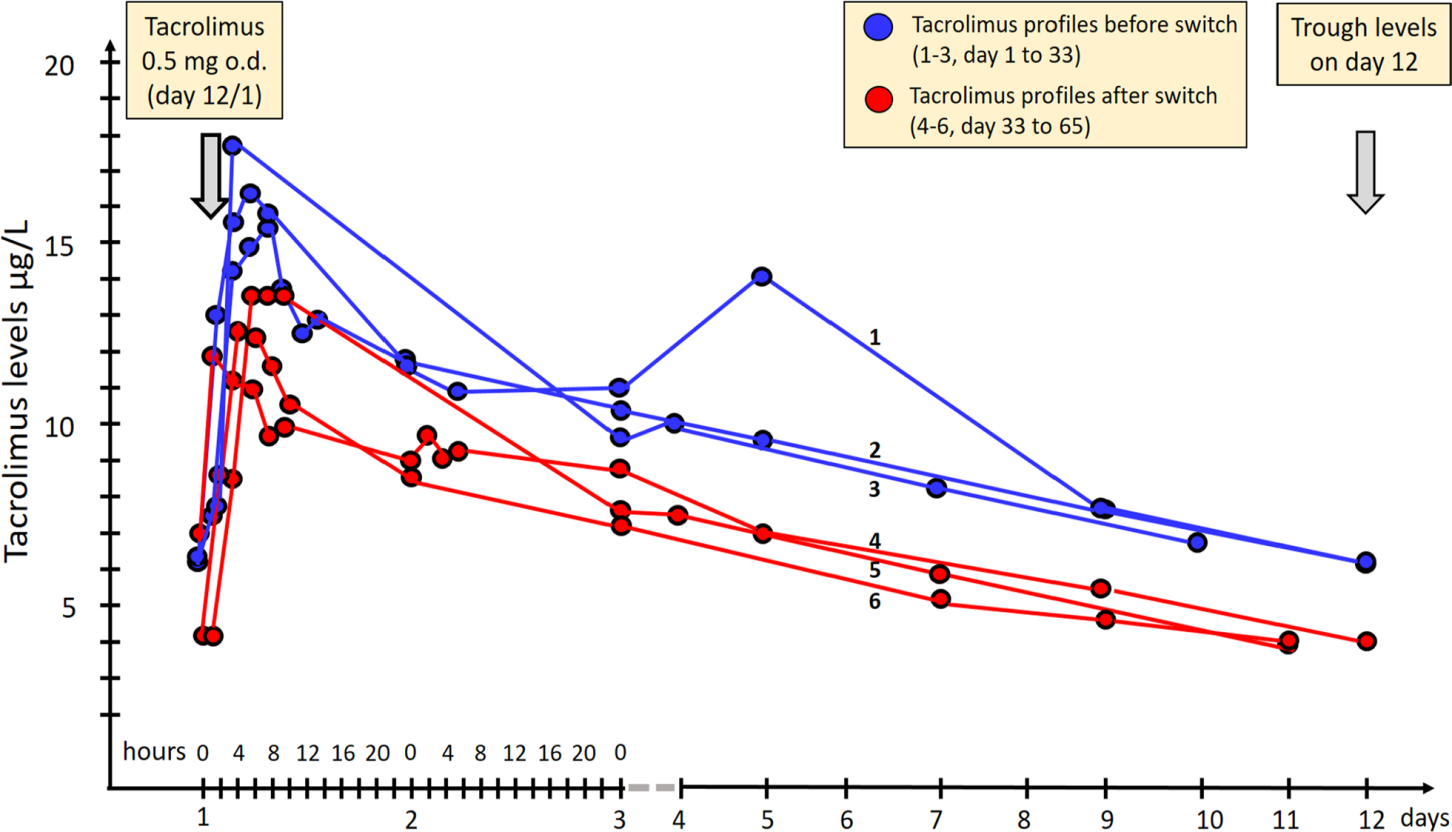
Tacrolimus profiles during ritonavir-boosted and cobicistat-boosted antiretroviral regimens. Three 11 days pharmacokinetic tacrolimus concentrations profiles in blue while on the ritonavir-boosted regimen before the switch and in red while on the cobicistat-boosted regimen after the switch. *For practical reasons (weekend, holidays) tacrolimus trough levels were measured on day 9 at the end of the third pharmacokinetic profile and on day 10 at the end of the sixth pharmacokinetic profile

**Fig. 2:**
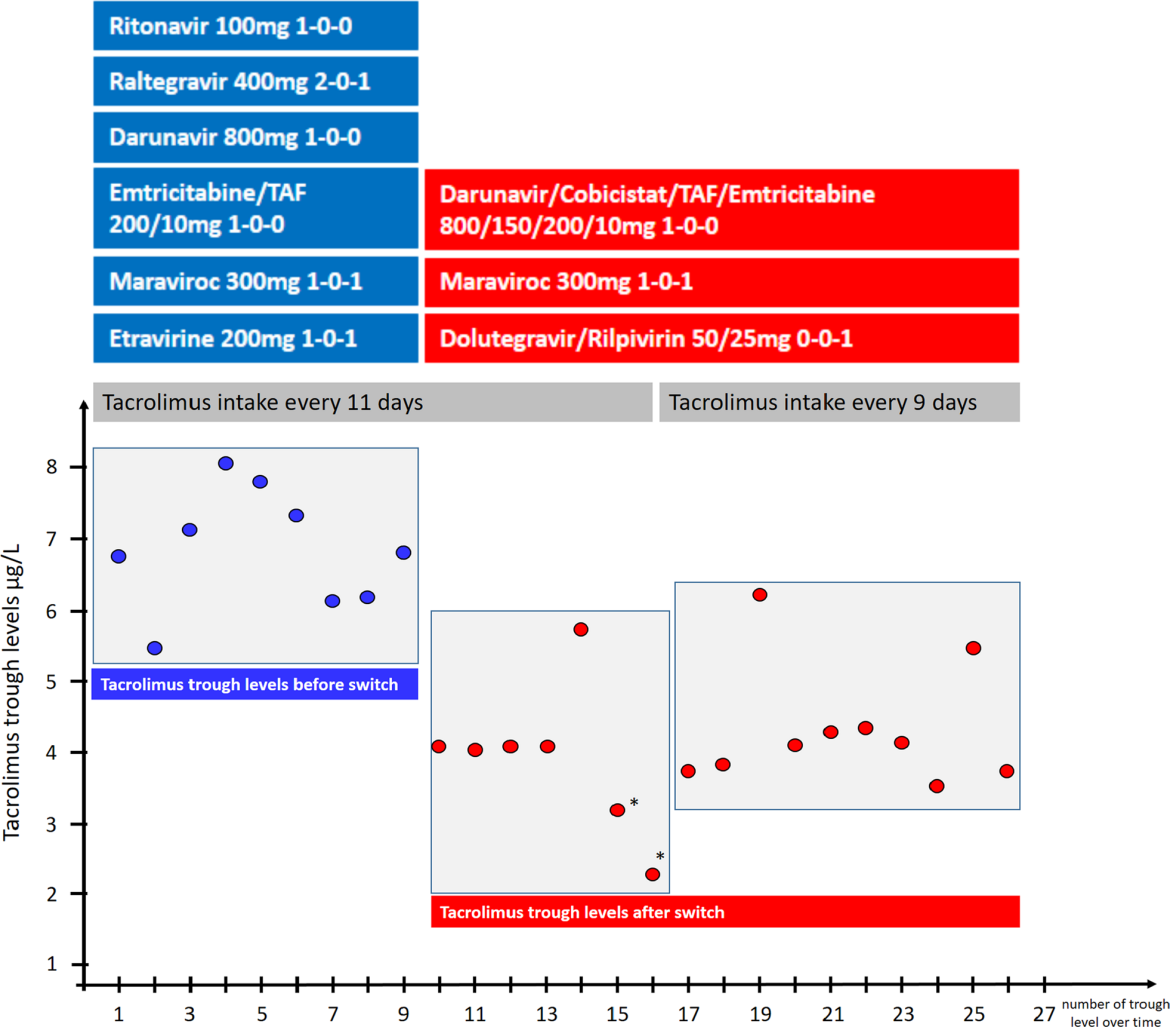
Antiretroviral treatment and tacrolimus trough levels before and after therapy switch. Blue bars indicate drugs before switch and blue dots tacrolimus levels before switch. Red bars indicate drugs after switch and red dots indicate tacrolimus trough levels after switch. Tacrolimus dosing interval was shortened from 11 to 9 days due to sub-therapeutic through levels*

Due to the high daily pill burden (i.e., 4 tablets mycophenolate mofetil, 10 tablets for the antiretroviral regimen, 7 pills for the treatment of cardiovascular risk factors, and 8 pills for additional indications), and with new combination antiretroviral pills available, a treatment simplification was envisaged. A feasible antiretroviral treatment considering the multi-drug resistant HIV virus was the combination of dolutegravir (INSTI), rilpivirine (NNRTI), maraviroc and the single tablet combination including emtricitabine, tenofovir alafenamide and darunavir boosted with cobicistat (darunavir/c). This modification allowed to reduce the daily number of pills for the antiretroviral regimen from 10 to 4. Due to the inducing properties of ritonavir, we predicted that a switch to cobicistat could result in higher tacrolimus concentrations. During three weeks before and three weeks after the switch we closely monitored tacrolimus drug levels (3 full pharmacokinetic profiles before and after changing the booster) to evaluate the effect on tacrolimus exposure (Figs. 1, 2). The pharmacokinetic parameters show a lower tacrolimus exposure after switching to cobicistat compared to ritonavir (Table 1; Fig. 1).

**Table 1:** Tacrolimus pharmacokinetic parameters when coadministered with darunavir/ritonavir (800/100 mg once daily) and with darunavir/cobicistat (800/150 mg once daily)

Pharmacokinetic parameter	1st pharmacokinetic profile	2nd pharmacokinetic profile	3rd pharmacokinetic profile
Tacrolimus co-administered with darunavir/ritonavir (800/100 mg once daily)			
C_0h_ (µg/L)	7.3	6.1	6.2^a^
C_max_ (µg/L)	15.1	17.7	17.6
T_max_ (h)	8	4	4
AUC_264h_ (µg h/L)*	2647	2385	2352^a^
T_1/2_ (h)	227	275	266
CL/F (L/h)	0.2	0.2	0.2
Tacrolimus co-administered with darunavir/cobicistat (800/150 mg once daily)			
C_0h_ (µg/L)	6.8^b^	4.1	4.0^a^
C_max_ (µg/L)	11.7	13.7	12.6
T_max_ (h)	2	8	4
AUC_264h_ (µg h/L)*	1747	1873	1235
T_1/2_ (h)	226	175	183
CL/F (L/h)	0.3	0.3	0.4

*The patient received tacrolimus once every 11 days. The area under the curve (AUC) represents a dosing interval of 11 days except for the third^a^ and sixth^a^ pharmacokinetic profile for which the dosing interval was 10 days for practical reasons. ^b^Trough level at the day of the switch, i.e., last trough level on darunavir/ritonavir (800/100 mg once daily) and immediately before administration of darunavir/cobicistat (800/150 mg once daily). AUC was calculated by the linear and logarithmic trapezoidal methods. C0h represents pre-dose plasma concentration and Cmax the maximum plasma concentration. Tmax is the time to reach Cmax and T1/2 indicates the elimination half-life. CL/F represents the apparent clearance calculated by dose divided by AUC. For comparison, tacrolimus pharmacokinetic parameters in renal transplant recipients in absence of strong inhibitors are: T1/2: 15.6 h; CL/F: 6.7 L/h^14^

Follow-up measurements showed a progressive decrease in tacrolimus trough levels reaching 2.1 µg/L eleven months post pharmacokinetic booster switch. As we aimed to reach trough levels of 3–5 µg/L in this patient with stable graft function 13 years after kidney transplantation, we accepted the decline in tacrolimus trough levels compared to pre-switch levels. However, we shortened the dosing interval from 11 to 9 days when trough levels declined below 3 µg/L, with trough levels thereafter ranging from 3.3 to 5.8 µg/L (Fig. 2).

The transient subtherapeutic trough levels had no adverse outcomes, in particular there was no deterioration of the renal function due to a rejection reaction (eGFR range 69–85 ml/min/1.7, creatinine range 89–101 μmmol/l, urine protein/creatinine ratio range 34–56 mg/mmol).

Mycophenolate mofetil trough levels remained stable in the therapeutic range without dose modification (before switch 0.4–2.3 mg/L; after switch 1.3–1.7 mg/L). Additionally, switching booster did not change the potassium levels (before switch 3.8–4.9 mmol/L and after switch 3.8–4.5 mmol/L,) or lactate (before switch 1.0–3.9 mmol/L and after switch 1.5–2.0 mmol/L), showing no consequences from the potential interaction between cobicistat (darunavir/c) and the comedication eplerenone and between dolutegravir (added in the regimen) and metformin (Additional file 1: Table S1). Under the new antiretroviral regimen, the HIV viral load remained mostly undetectable and the patient did not experience any adverse effects due the new cART.

## Discussion

In this follow up case report [8] we describe the impact of a switch in pharmacokinetic booster on tacrolimus levels in a HIV-infected kidney transplant recipient.

While the literature has reported on many cases of DDIs between ritonavir-boosted PIs and immunosuppressants, only few case reports investigated the interaction of cobicistat boosted PIs and tacrolimus and also found important DDI, albeit not always identical to ritonavir [12–14].

Our long-term case study documents well that the pharmacokinetic booster in a PI-based antiretroviral regimen profoundly impacts tacrolimus levels. In this patient, 15 years ago, the introduction of a boosted PI (darunavir/r) was challenging due to the strong inhibition of CYP450 3A4, requiring a substantial dose reduction and extension of the dosing interval of the calcineurin-inhibitor [8]. In the present situation, the replacement of the booster from ritonavir to cobicistat could be managed by gently adjusting the dosing interval but necessitated close monitoring.

We expected that a switch to cobicistat would result in an increase in tacrolimus levels given that, unlike ritonavir, cobicistat has no inducing properties on intestinal P-glycoprotein thereby resulting in a higher absorption of tacrolimus. Against our expectation the switch resulted in lower tacrolimus levels. Furthermore, the patient was switched from etravirine to rilpivirine which should also lead to an increase in tacrolimus levels given that etravirine is an inducer of CYP3A4 whereas rilpivirine has no effects on CYP3A4. We excluded possible other causes for the decrease in tacrolimus levels. The patient did not receive co-medications with inducing properties, did not have episodes of diarrhea or vomiting and did not present an increase in body weight. Furthermore, no changes were observed in the liver or kidney function. Finally, the patient was maintained on the same tacrolimus formulation, did not change his drug intake habits (i.e., tacrolimus bioavailability is modified by food) [15] and reported an excellent adherence. The decrease in tacrolimus levels was unexpected and therefore underlines the importance of closely monitoring drug levels when changing antiretroviral treatment including the pharmacokinetic booster. While our patient was in a stable long-term phase after transplantation, at early stages, the risk of rejection or acute calcineurin-inhibitor toxicity could be increased even with minor changes in calcineurin-inhibitor drug levels.

To the best of our knowledge, this is the first report on the impact of a change in the pharmacokinetic booster on tacrolimus levels. Our report underlines that cobicistat and ritonavir are not fully interchangeable and might require an adaption of the calcineurin-inhibitor dose and/or dosing interval. Under close drug monitoring, the introduction of a cobicistat-based regimen in patients on ritonavir-boosted PI regimen is safe and, in our patient, allowed a relevant reduction of the pill burden without adverse events. Since transplantation has become the standard of care for PLWH with end-stage liver or kidney disease [3], this framework will probably challenge an increasing number of clinicians in the future. Close therapeutic drug monitoring, as well as clinical follow up and good adherence to the medications are critical to successfully manage these complex situations.

## Supplementary Information

**Additional file 1: Table S1**. Co-medication.

## Data Availability

Due to the nature of a case report we show all relevant information in the manuscript. No additional data repository is foreseen.
