# Supplementary material for: Switch from a ritonavir to a cobicistat containing antiretroviral regimen and impact on tacrolimus levels in a kidney transplant recipient
Source: Virol J. 2023 May 5;20:89. doi: 10.1186/s12985-023-02058-3 (PMC10163738; doi:10.1186/s12985-023-02058-3)
Supplement: Supplementary file 1 — Additional file 1: Table S1. Co-medication. [file 12985_2023_2058_MOESM1_ESM.docx]

**Supplementary Table**

**Table S1: Co-Medication**

| **Co-Medication before switch** | **Co-Medication after switch** |
| --- | --- |
| Mychophenolat-Mofetil 750mg 1-0-1 | Mychophenolat-Mofetil 750mg 1-0-1 |
| Tacrolimus 0.5mg every 11 days | Tacrolimus 0.5mg every 11 days |
| Sitagliptin/Metformin 50/1000mg 1-0-1 | Sitagliptin/Metformin 50/1000mg 1-0-1 |
| Eplerenone 25mg 2-0-0 | Eplerenone 25mg 2-0-0 |
| Cerivastatin 10mg 1-0-0 | Cerivastatin 10mg 1-0-0 |
| Amlodipin 5mg 1-0-0 | Amlodipin 5mg 1-0-0 |
| Aspirin 100mg 1-0-0 | Aspirin 100mg 1-0-0 |
| Olmesartan 20mg 1-0-0 | Olmesartan 20mg 1-0-0 |
| Oxycodon-Naloxon 5/2.5mg 1-0-1 | Oxycodon-Naloxon 5/2.5mg 1-0-1 |
| Testosteron Gel 20mg/g 4 strokes/d | Testosteron Gel 20mg/g 4 strokes/d |
| Trimipramin 25mg 0-0-1 | Trimipramin 25mg 0-0-1 |
| Magnesium 5mmol 1-0-1 | Magnesium 5mmol 1-0-1 |
| Vitamin D3 1000 IE 1-0-0 | Vitamin D3 1000 IE 1-0-0 |
| Insulin s.c. | Insulin s.c. |
